# Efficient Usage of Dense GNSS Networks in Central Europe for the Visualization and Investigation of Ionospheric TEC Variations

**DOI:** 10.3390/s17102298

**Published:** 2017-10-10

**Authors:** Grzegorz Nykiel, Yevgen M. Zanimonskiy, Yuri M. Yampolski, Mariusz Figurski

**Affiliations:** 1 Faculty of Civil and Environmental Engineering, Gdansk University of Technology, 80-233 Gdańsk, Poland; mariusz.figurski@pg.edu.pl; 2 Institute of Radio Astronomy, National Academy of Sciences of Ukraine, 61002 Kharkiv, Ukraine; zanimonskiy@rian.kharkov.ua (Y.M.Z.); yampol@rian.kharkov.ua (Y.M.Y.)

**Keywords:** ionospheric disturbances, GNSS, TEC variation maps, neutral wind, geomagnetic storm

## Abstract

The technique of the orthogonal projection of ionosphere electronic content variations for mapping total electron content (TEC) allows us to visualize ionospheric irregularities. For the reconstruction of global ionospheric characteristics, numerous global navigation satellite system (GNSS) receivers located in different regions of the Earth are used as sensors. We used dense GNSS networks in central Europe to detect and investigate a special type of plasma inhomogeneities, called travelling ionospheric disturbances (TID). Such use of GNSS sensors allows us to reconstruct the main TID parameters, such as spatial dimensions, velocities, and directions of their movement. The paper gives examples of the restoration of dynamic characteristics of ionospheric irregularities for quiet and disturbed geophysical conditions. Special attention is paid to the dynamics of ionospheric disturbances stimulated by the magnetic storms of two St. Patrick’s Days (17 March 2013 and 2015). Additional opportunities for the remote sensing of the ionosphere with the use of dense regional networks of GNSS receiving sensors have been noted too.

## 1. Introduction

Multiposition radio sounding of near Earth plasma by signals of the global navigation satellite system (GNSS) can be effectively used for studying and simulating ionospheric processes. This became possible due to the development of networks of dual-frequency receiving sensors that were originally designed primarily for solving navigation and geodetic tasks. Such studies use different techniques for data processing and exploit GNSS receivers located both on the Earth’s surface and on low-orbit satellites. Maps of total electron content (TEC) are the most widely used representation for the structure of the ionosphere [1,2,3,4,5,6].

The global ionospheric maps (GIMs) of TEC available from the Internet are a unique source of information on a large-scale about the ionosphere’s structure and dynamics [1]. The imperfections of GIMs are low temporal and spatial resolutions of about one hour and hundreds of kilometers, respectively [2]. To reduce the first parameter down to several minutes, the technique of regional TEC map reconstruction has been developed and implemented for different areas of the globe. At the same time, the spatial resolution for most practical implementations of regional TEC maps is the same as for the global ones [2,3,4,7]. Investigations of TEC variations associated with powerful processes in the atmosphere [8,9,10,11,12,13,14,15] were done by using the data of vast continental-scale networks of GNSS receivers. Results for the visualization of processes that occurred in the near Earth space were given for groups of ionospheric pierce points only. The techniques used in the cited works permit us to determine the parameters of ionospheric inhomogeneity, but do not allow us to build real maps interpolated to a regular grid.

An increased interest in the diagnostics of Atmospheric Gravity Waves (AGW)/travelling ionospheric disturbances (TID) in recent years can be attributed to a number of reasons. First, their origin and propagation are beyond the scope of modern predictive models of the dynamics of the Earth’s gas envelope. At the same time, variations of the electron density of near Earth plasma caused by the propagation of these waves reach up to tens of percents and may cause significant disturbances to terrestrial and space-borne transionospheric radio-communication systems. Second, AGW are normal atmospheric waves, which can propagate with low attenuation over long distances both horizontally and vertically. Correspondingly, AGW/TID play the part of a transport agent, which carries a perturbation from the troposphere to the thermospheric heights, thus providing energy exchange in the Earth’s surface–atmosphere–ionosphere system. Moreover, as has been shown in [11], AGW propagation at dynamo region altitudes leads to a modulation of the magnetic field of the Earth, not only in the region of their location, but through all the magnetic tube up to the magneto-conjugation region. Finally, AGW/TID contain information about the sources of their generation. The natural sources of these waves are powerful weather fronts, hurricanes, typhoons, earthquakes, volcanic eruptions, forest fires, magnetic storms, the precipitation of energetic particles in cusp areas, strong lightning discharges, etc. Due to the increased energy output of modern civilization, the artificial sources of AGW/TID are powerful military and industrial explosions, missile launches, emergency heat, and radiation and chemical emissions.

AGW/TID modulate the electron density in the horizontal direction, exciting quasi-periodic variations in TEC [12,13,14,15]. Major characteristics of such variations are the wavelength and direction of propagation in the space domain, as well as the temporal period. The results presented in this paper show the ability to reconstruct the fine spatio-temporal structure of AGW/TID using a proposed original technique for the processing of TEC records provided by a dense network of ground-based GNSS receivers.

The study of the fast dynamic processes of plasma irregularities of medium scale, from tens to hundreds of kilometers, requires a better spatio-temporal resolution of TEC maps than that of the well-known global ones [16]. In some countries, significant efforts have been made to develop methods of constructing regional TEC maps with a resolution of about 50 km in space, which, however, have not yielded any tangible effect yet. Although the maps given in available sources [2,6,7,16] have a small step of a regular grid of points, the data they contain is not as detailed as the resolution indicates.

The aim of our study is to investigate the spatial structure and temporal dynamics of variations in the TEC of the ionosphere at middle latitudes using the developed method [17] for mapping and measuring the parameters of TIDs. Based on the signals from near zenith satellites received by 670 GNSS stations located in central Europe, we estimated high spatial (0.1° × 0.1°) and temporal (30 s) maps of TEC variations, which can be used to provide detailed analyses of TEC structures and their parameters, such as direction and velocity.

## 2. Materials and Methods

**Figure 1:**
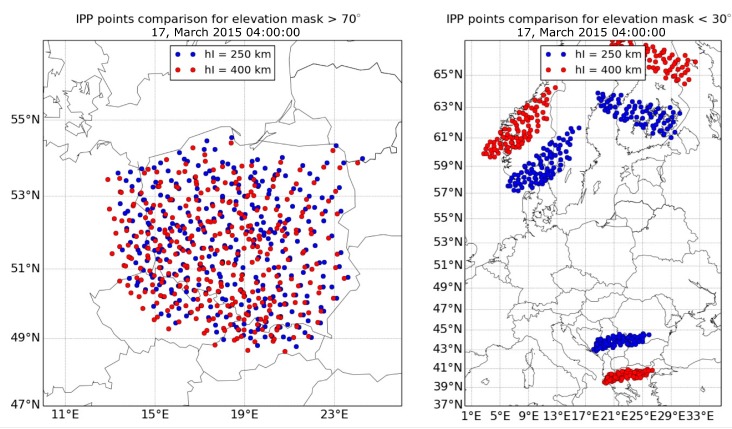
Comparison of ionospheric pierce points (IPPs) distribution for 250 km (blue) and 400 km (red) ionospheric layer heights and for elevation masks >70° (**left**, one satellite) and <30° (**right**, three satellites).

Traditionally, TEC maps suggest the use of a thin layer model of the ionosphere. Total electron content calculated according to the GNSS receivers’ data refers to the line of sight of a “satellite–receiver” characterized by elevation and azimuth. To display the TEC as a geographical map, current values are projected onto a spherical surface, which simulates the ionosphere in the form of a thin layer [18,19]. Ambiguity in the choice of a spherical shell’s height is a source of uncertainty in coordinates of ionospheric pierce points (IPPs), whose position relative to different satellites and observation points varies depending on the selected layer height. This leads to the fact that local maps, based on several observed satellites’ data, depend strongly on the choice of the height of the ionospheric layer. In Figure 1, a comparison of IPPs distributions between two heights of the ionospheric layer, including different elevation angles, is shown. Here, the data observed from 125 GNSS reference stations from the dense Polish nationwide network ASG-EUPOS were used [20]. On the right, IPPs for satellites with elevation angles below 30° are shown. Their IPPs were located at a large distance from each other, reaching 28°. However, these distances depended on the adopted ionospheric layer height. Of course, in the presented case, only the low elevation angle satellites were used, but our intention was to show the difficulties of using these data in ionospheric modeling and possible uncertainty in any interpretation of the models obtained. For the whole day, the observed distance differences reached up to 6° and 3° for the longitude and latitude directions, respectively. For comparison, the IPPs for satellites with elevation angles over 70° are shown on the left side of Figure 1. In this case, it can be seen that IPPs cover the entire area of Poland and the differences due to the adopted layer height were much smaller. The maximum obtained differences never exceeded 0.5° and 0.3° in the longitude and latitude directions, respectively. The use of high elevation angle satellites allows us to obtain an ionospheric variation model immediately over the selected area and without the large errors associated with an incorrectly taken layer height. It is quite natural that there is no possibility to use this advantage at high latitudes. A high spatial resolution of the IPPs can be obtained by using the dense regional networks of GNSS stations, such as ASG-EUPOS, SAPOS, etc.

**Figure 2:**
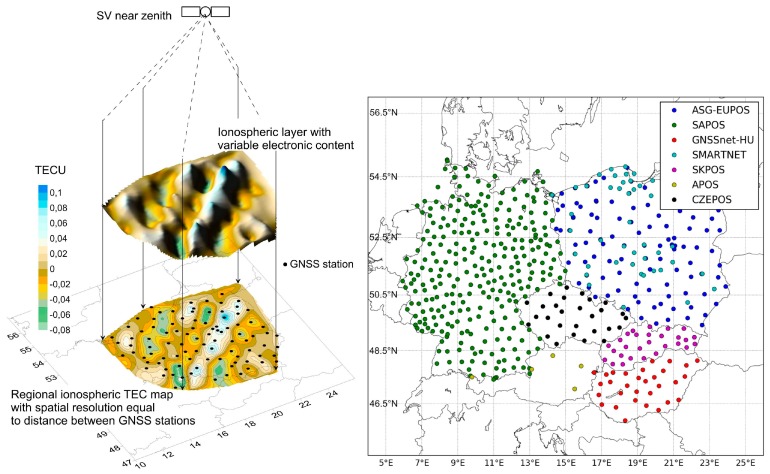
Scheme of the total electron content (TEC) mapping method of orthogonal parallel-beam projection (**left**) and the global positioning system (GPS) reference stations used in this study (**right**). GNSS: global navigation satellite system.

Based on the above comparison, we used only the observations from the quasi-vertical direction, using data from the near-zenith satellites (elevation angle more than 70°). In this case, total electron content is measured practically directly above the receivers, and the spatial resolution is determined by the distance between them. This method of the orthogonal projection of variations in the electron content of the ionosphere (OPVECI) [17] is similar to the orthogonal parallel-beam projection used in medical radiography (Figure 2, left). Such a solution has enabled us to achieve three main effects. First, the distortion of maps in a thin layer model due to an ambiguity of layer height does not occur because the spatial patterns of IPPs obtained in a single satellite (space vehicle, SV) scheme have almost the same appearance at altitudes ranging within 100 to 450 km. Second, the models of ionospheric disturbances are obtained directly over the area of interest. The third effect is related to the TEC value’s calculation. In the presented solutions, a geometry-free linear combination was used to estimate a slant total electron content (STEC): the linear integral of the electron density along the line of sight toward the satellite in each epoch t. STEC is expressed in TEC units, defined as TECU = 10^16^ el/m^2^.

In order to obtain the vertical value of TEC, the STEC should be converted using the simple mapping function cos z’ [5]:

TEC_t_ = cos(z’)STEC_t_ (1)

where z’ is the reduced zenith distance of satellites at the ionosphere layer’s height.

However, in the presented solutions, only satellites with a high elevation angle were used, so the value of the mapping function is close to a unity that generates small differences between TEC values in the slant and zenith directions.

However, such a solution does not eliminate a constant and large error (bias) caused by the unknown value of a phase’s ambiguity. In order to eliminate it, 3-degree polynomials were fitted into the obtained time series of STEC. After subtracting the fitted polynomial from the raw STEC values, variations of STEC (ΔSTEC) were obtained. Because the differences between TEC and STEC values are small, later we use only the term ΔTEC to mean variations of TEC. After processing the data for all stations and satellites, an evenly spaced grid mesh was created by employing the nearest neighbor method. A grid file is used to create a map and a three-dimensional shaded rendering. The height of the three-dimensional (3D)-surface corresponds to the ΔTEC value of the associated grid node. Often, a 3D-image effectively complements the map for a more convenient interpretation of the spatial distribution (Supplementary movies).

The use of a large number of closely spaced stations allows us to obtain ionospheric variation maps with a very high spatial resolution, being 0.1° × 0.1°. Moreover, the mapping occurs without time averaging, thus temporal resolution is determined by the sampling rate of the receivers (30 s in this study). The generated maps and 3D surfaces visualize the results of processes in the ionosphere and magnetosphere and can be important sources of information for solving inverse problems of modeling the causes of ionospheric variations. The TEC variation maps are, in fact, two-dimensional pictures, for the study of which you can use well-designed and powerful image analysis techniques [21] as well as cartographic methods [22].

In the present study, we considered TEC variation (ΔTEC, Figure 2, left) maps as measurements of parameters of ionospheric irregularities by employing global positioning system (GPS) observations from several dense national-wide networks in central Europe [23]. For this, we used data from the following networks (Figure 2, right): ASG-EUPOS and SMARTNET (Poland), SAPOS (Germany), SKPOS (Slovakia), CZEPOS (Czech Republic), GNSSnet-HU (Hungary), and APOS (Austria), all of which belong to the EUREF Permanent Network (EPN). The total number of used GNSS stations amounted to 670 in 2015.

**Figure 3:**
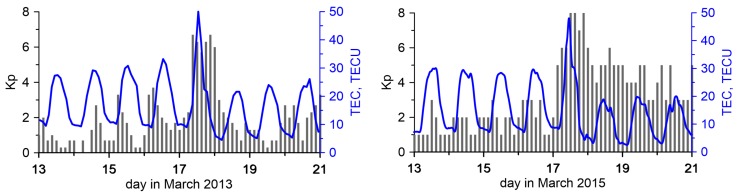
Planetary K index estimated for 13–20 March 2013 (**left**) and 13–20 March 2015 (**right**) and change of TEC values over central Europe during the analyzed time intervals.

Here, the results obtained for the immediate vicinity of two stormy days are given: 13–20 March 2013 and 13–20 March 2015. For both cases, in Figure 3, the estimated Kp indexes are given [24]. From this Figure, it can be seen that in 2013 the disturbances occurred only on the 17th of March and the Kp index value was near 6. Definitely, higher and longer disturbances were observed in 2015. The geomagnetic storm began on 17 March 2015 and continued until the middle of 19 March 2015. The strongest disturbances occurred, similarly as in 2013, on 17 March 2015. However, their values were much higher and amounted to a maximum Kp of 8.

It will also be observed that after the active phase of the geomagnetic storms, for several days the TEC’s variation was smaller than in the days preceding the perturbation. Apparently, this is due to a TEC decrease in the recovery phase of the ionosphere after the geomagnetic disturbance. This result is consistent with the conclusions made by, e.g., [25]. Figure 3 shows the change in TEC according to the GIMs [1] over central Europe during the analyzed time intervals in 2013 and in 2015. The fact of the proportionality of TEC variations and the absolute value of TEC is noted in [14].

This surprising coincidence that on the same day of different years geomagnetic storms have occurred has already been noticed by some other authors, e.g., [25]. They found, based on TEC values estimated from GPS observations, that the ionosphere’s behavior during these two geomagnetic storms was similar. In both cases, a TEC increase occurred during the main phases of the geomagnetic storms. These increases were followed by a TEC decrease during the recovery phase. In the event of 2015, the TEC’s increase during the main phase had more intensity even though it was of the same duration as that of 2013. However, the intensity and duration of the TEC’s decrease was larger in 2015 than in 2013.

## 3. Results: Regional Maps of Travelling Ionospheric Disturbances at Low and High Geomagnetic Activity

In this Section, local maps are analyzed to retrieve travelling ionospheric disturbances. To obtain more valuable data, which can derive all of the information about the spatial distribution of irregularities and their parameters, the data for quiet and disturbed days were used (from the 13th to the 20th of March).

The sequences of TEC variation maps and the corresponding two-dimensional spatial autocorrelation functions were the object of processing by cartographic analysis methods [26].

**Figure 4:**
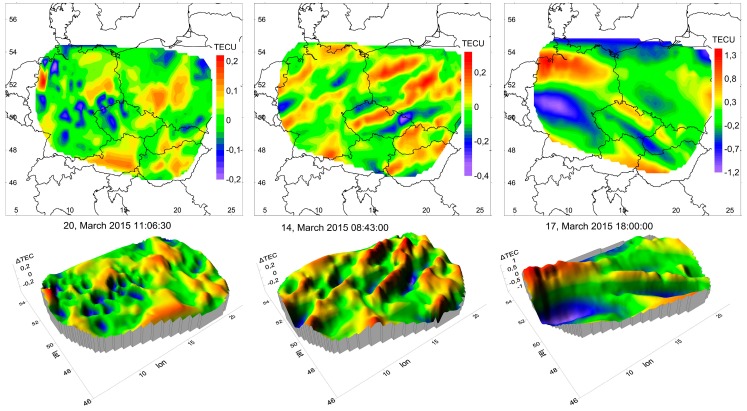
Representative examples of TEC variation spatial distribution.

It turns out that TEC irregularities are present at any time and at any geomagnetic activity. They can be divided into at least three categories. In Figure 4, examples of TEC variations’ spatial distribution are shown in the form of maps and 3D-surfaces: random background variations (Figure 4, left), quasi-deterministic wave-like structures (Figure 4, center), and space partitioned ones, which occur during a geomagnetic storm’s active phase (Figure 4, right). Background variations have an autocorrelation function corresponding to a random two-dimensional field. It is possible, however, that the TEC variations shown in Figure 4, left are not entirely random and can correspond to the model presented in the paper [27].

Quasi-wave variations have an obvious wavy structure of the spatial distribution of TEC variations, and, accordingly, a wave-like form of spatial autocorrelation. These structures slightly change their shape over tens of minutes. Space partitioned structures are characterized by the fact of presence on the map simultaneously of two or more relatively stable segments with essentially different properties.

**Figure 5:**
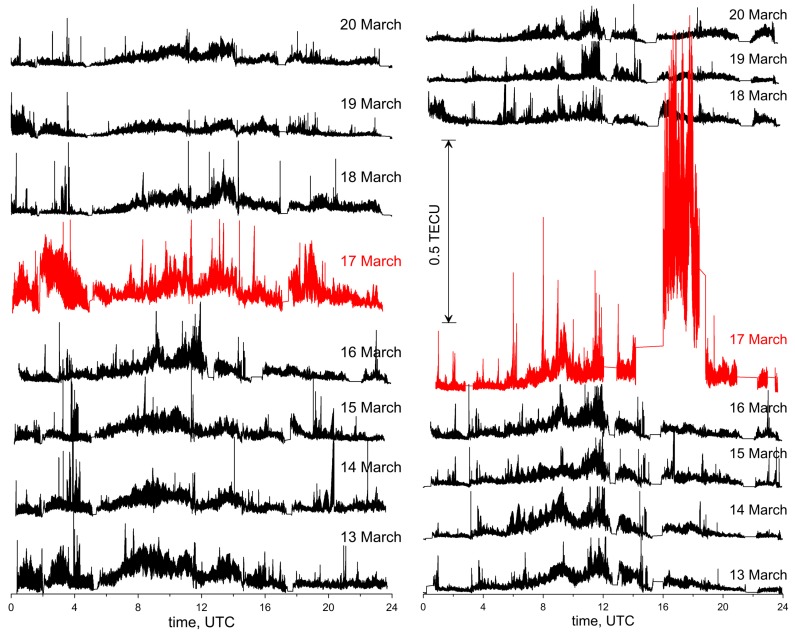
RMS of TEC variations in diurnal cycles: year 2013 (**left**) and 2015 (**right**).

For the 16 days analyzed, the root mean square (RMS) of TEC variations in diurnal cycles throughout the maps were calculated and are shown in Figure 5. The regular dependence of the RMS value on the time of day can be clearly seen. The highest values are obtained for the hours about mid-day. This is, of course, caused by the higher level of TEC at this time and it is normal for the quiet days. However, the situation changes when the geomagnetic storms occur. A significant increase of RMS during the geomagnetic storms can be noticed. This is consistent with the conclusions made by, e.g., [28]. 

On 17 March 2015, its values between 16 and 19 UTC (Coordinated Universal Time) amounted to more than 1 TECU. For comparison, on the quiet days, the RMS values were below 0.1 TECU for the whole day. From the given results, it can also be seen that the geomagnetic storm in 2013 had a much lower intensity than the one in 2015.

**Figure 6:**
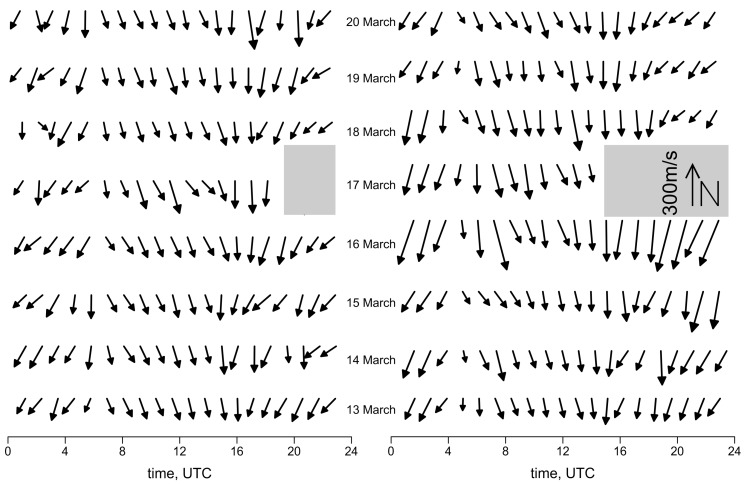
Variations in the speed and direction of motion of the background ionospheric irregularities in the diurnal cycle: year 2013 (**left**) and 2015 (**right**). Two gray zones mask the uncertain rating of direction and speed during the storms. In the right zone, the legend for the speed vector is shown.

Figure 6 shows the variation of speed and direction of movement in daily cycles. To determine the parameters of these moving background heterogeneities, a well-known method for processing the images of moving objects was used. First, two-dimensional cross-correlation functions of pairs of successive maps are calculated. Lags in latitude and longitude, corresponding to the maximum of the cross-correlation function, are an estimate of the displacement of the spatial structure of TEC variations over the time between two consecutive maps: in our case, 30 s. Thus, the velocity components are calculated. In addition, lastly, the time series of the velocity components are smoothed to reduce random error and ultimately undergo thinning for image convenience.

It can be stated that background heterogeneity moves in what is a more or less regular manner. The direction and speed of the movement are very similar during the quiet days in 2013 and 2015. A speed increase was observed for both of the geomagnetic storms that occurred on the 17th of March of those two years. Parameters of motion of irregularities on the maps as a whole could not be obtained when the main phase of the geomagnetic storms occurred. This situation is caused by the very complex, ambiguous structures of irregularities. The results of the special processing of maps in these cases are given below.

**Figure 7:**
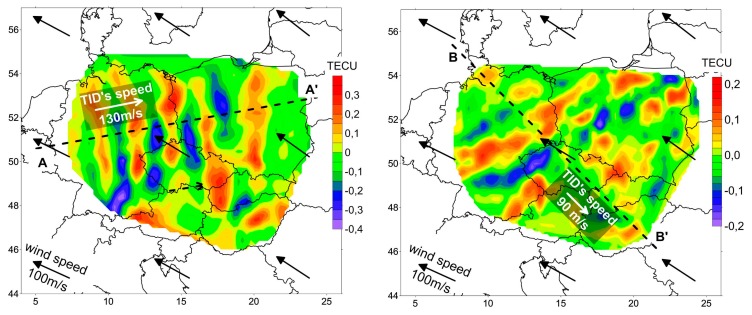
Map of TEC variations over investigated region of 13, March 2013 at 08h50m (**left**) and of 13, March 2015 at 08h50m (**right**).

On the background of the ever-present disturbances, several times a day, quasi-deterministic wave-like variations appear. So far, on a small, generally speaking, statistical basis, it is shown that the form and parameters of these variations, as well as the time of occurrence, are repeated from day to day and even from year to year. Figure 7 illustrates this interesting situation, the cause of which is still waiting for its study.

Figure 7 shows black and white arrows. The first of these (black) show the direction and speed of thermospheric wind at the height of the maximum of the F2 ionospheric layer. We have used the horizontal wind model (HWM-07) [29], which is a commonly used model available in the MATLAB software package. The second (white) show the direction and speed of TIDs estimated for the obtained TEC variation models. It can be seen that on 13 March 2013 the speed of ionospheric disturbances was about 130 m/s, with an eastern direction. Similar speed values were calculated for the thermospheric wind from the model, but with an almost opposite direction. In Figure 7, the results from the 2015 year for the same time are shown too. It can be seen that the wind speed has a similar direction and speed as in 2013. However, it can also be noticed that the investigated TID’s speed is smaller, and the direction changes from the east to southeast.

The TIDs in both cases are seen in the supplementary movies S1–S4.

To characterize the detected structures, spatial autocorrelation functions (ACFs) were constructed for map segments including wavy zones. The size of the segments ranged from the entire map to one period of the undulating structure. It is supposed that the spatial size of the structure corresponds to the size of the segment of the most variable ACF that consequently will be used in the subsequent analysis. In both cases, TIDs were detected over a territory of about 4105 km^2^. It should be noted that the possibility for determining the size of the TID zone appears to be due to the usage of the described method of visualization.

**Figure 8:**
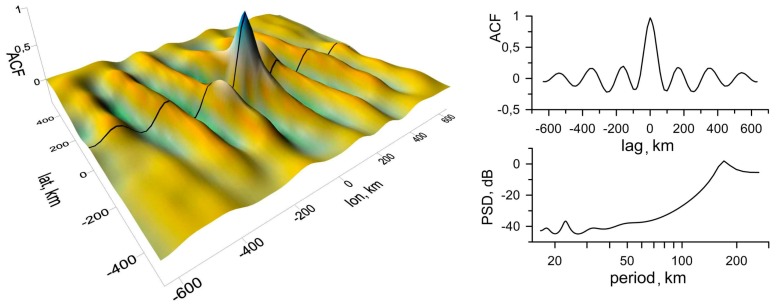
Spatial autocorrelation function (ACF) of TEC variations on 13 March 2013 at 08h50m (**left**), its central profile and periodogram (**right**).

**Figure 9:**
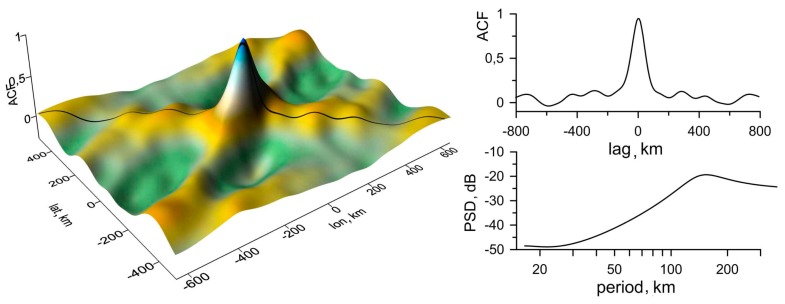
Spatial ACF of TEC variations on 13 March 2015 at 08h50m (**left**), its central profile and periodogram (**right**).

Most of the variable ACFs discussed above are shown in Figure 8 (left) for year 2013 and in Figure 9 (left) for year 2015. The quantitative parameters of a wavy structure are determined in the cross-section (the central profile) of an ACF and in the corresponding power spectral density (PSD) plots in Figure 8 (right) and in Figure 9 (right). In both cases, the modulation periods are estimated to be 170 km. To trace a selected TEC structure’s time history, consecutive maps constructed at one-minute intervals were analyzed during whole time of the TID occurring. The direction of a TID’s propagation (along the lines A-A’ and B-B’, respectively) is orthogonal to the wave front. The phase velocity is determined from the set of cross-sections of the spatial distribution of TEC variations along a certain direction.

**Figure 10:**
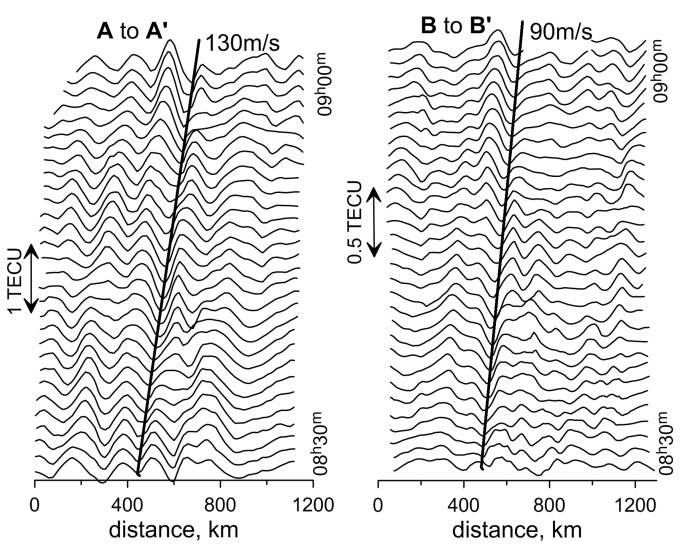
Profiles of TEC variations along the line A-A’ in Figure 7 from 08h30m to 09h00m on 13 March 2013 (**left**) and along the line B-B’ from 08h30m to 09h00m on 13 March 2015 (**right**), in the form of a “waterfall”. The bold line shows the wave pattern trajectory.

The left panel of Figure 10 shows the change in the profile of TEC variations along the A-A’ line in Figure 7, left (from 08h30m to 09h00m on 13 March 2013) in the form of a “waterfall”. Changes in the structure were analyzed on the maps constructed at intervals of one minute. A similar change in the profile of TEC variations is shown in Figure 10, right, for the line B-B’ in Figure 7, right (from 08h30m to 09h00m on 13 March 2015). These figures can be interpreted as a propagation in the ionosphere of the quasi-wave disturbance, which moves eastward at a speed of about 130 m/s (in 2013) and southeastward at a speed of 90 m/s (in 2015). In both cases, these movements in a stable manner are observed for more than half an hour.

**Figure 11:**
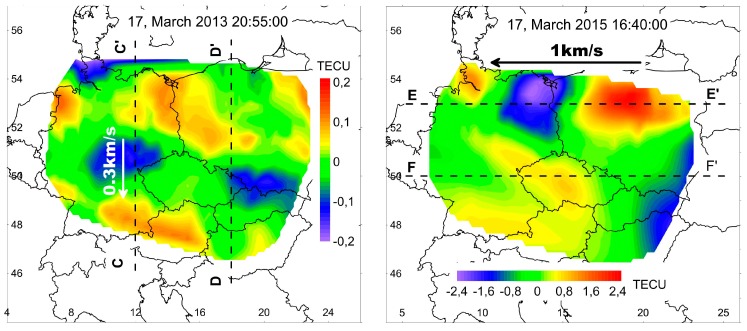
Space partitioned structures, which occurred during the geomagnetic storms' active phase. Maps of TEC variations over investigated region on 17 March 2013 at 20h55m (**left**) and on 17 March 2015 at 16h40m (**right**).

Maps obtained during the geomagnetic storms’ active phases were analyzed in a special way. We investigated sequences of maps using methods of general scene analysis [21], which is based on dividing each picture into regions representing different or differently changing structures. In both cases, two zones with different parameters of motion of inhomogeneities can be selected (Figure 11). These zones are seen in the supplementary movies S3 and S4 during 10-min intervals.

**Figure 12:**
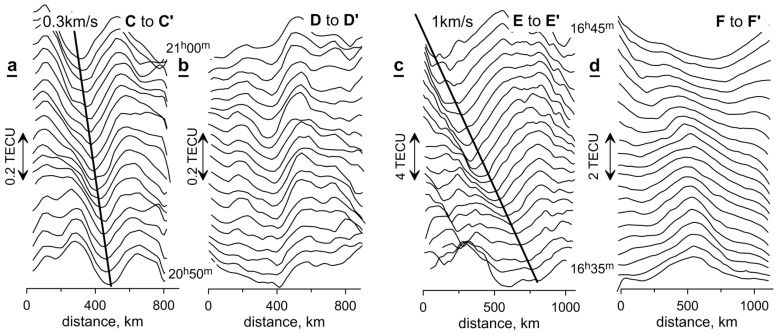
Profiles of TEC variations, in zones with (**a**, **c**) as well without (**b**, **d**) active travelling disturbances, along the lines C-C’ and D-D’ in Figure 8 left on 17 March 2013 (**a**, **b**) and along the lines E-E’ and F-F’ in Figure 8 right on 17 March 2015 (**c**, **d**), in the form of a “waterfall”. The bold lines show the trajectory of the disturbances.

Figure 12 is identical to Figure 10. It shows the changes in the profiles of TEC variations along the lines C-C’, D-D’, E-E’, and F-F’ that represent the directions with minimal transformations of spatial structures during 10 min.

Disturbances in 2013 were characterized by a somewhat greater level of TEC variations than during quiet days. At the main phase of the storm to the West of 15° E, there was a steady movement southward at a speed near 0.3 km/s (Figure 12a). To the East of 15° E, a structure similar to a soliton remains motionless for a dozen minutes (Figure 12b).

Disturbances in the main phase of the storm in 2015 were characterized by TEC variations being more than an order of magnitude greater than on the quiet days. The zone with the most intense variations of TEC was located to the north of 52° N. The disturbances apparently moved with a speed near 1 km/s, being significantly higher than the speed of sound (Figure 12c). An understanding of the drivers of this effect, however, remains a challenge due to the complex interactions between the possible mechanisms [30]. To the south of 52° N, the variations of TEC are of a lower intensity, but they change their spatial shape more rapidly and therefore it is not possible to reliably measure the speed of their movement (Figure 12d).

## 4. Discussion and Conclusions

In this paper, we present a technique and processing algorithms based on the data of the dense continental-wide network of GNSS receivers for the visualization of processes in the ionosphere and magnetosphere, which are the source of variations of the total electron content between satellites and ground receivers.

The possibility to build maps of TEC variations with the resolution of tens of kilometers with the temporal rate of tens of seconds is demonstrated. This allows us to analyze the structure and temporal evolution of mesoscale ionospheric irregularities.

In accordance with current trends in the development of a four-dimensional (4D) geodesy, the use of the proposed methodology makes it possible to obtain and effectively use a large amount of information organized in the form of time sequences of maps. The paper shows the possibility of using image processing methods and cartographic analysis for the study and parametrization of ionospheric processes.

Our statistics of processes in the quiet ionosphere are still little. Nevertheless, it is shown that undulating TIDs occur regularly in the ionosphere at approximately the same time of day and are likely related to the thermospheric wind at ionospheric heights. The measured parameters of TIDs are consistent with the literature data. For the first time, the duration of the existence and the area occupied by a stable TID were estimated.

On the maps that were obtained during the unique events of the geomagnetic storms on 17 March 2013 and 2015, the variations of TEC form very peculiar structures with a dynamics essentially different from the dynamics of ionospheric processes on quiet days. Fortunately, in the arsenal of image processing methods, there are opportunities for a reasonable segmentation of the spatial picture. For the first time, we were able to determine the quantitative TID parameters simultaneously on two parts of the TEC variation map over central Europe.

During a powerful geomagnetic storm on 17 March 2015, in the ionosphere above the central European region, a sharp latitudinal boundary of the increase in the intensity of TEC variations during the main phase of the storm was found, located at approximately 52° N. The level of variations of the TEC north of this boundary exceeds the background values by more than an order of magnitude. At this time, the main direction of the motion of the aperiodic inhomogeneities was zonal in nature from east to west with a supersonic velocity of about 1 km/s.

Further studies of visualized structures at different stages of geomagnetic storms are very intensive and yield interesting results, which discussion is beyond the scope of this publication.

Taking into account the existence and intensive development of dense networks of GNSS receivers in different regions of the globe and the simplicity of the proposed method for the visualization of TEC variations with high spatial–temporal resolution, this approach can be used for a more detailed investigation of ionospheric processes on a planetary scale.

## Supplementary Materials

The following are available online at http://www.mdpi.com/1424-8220/17/10/2298/s1, Animations of the TEC maps during four events can be viewed in supplementary movies, S1: 13 March 2013 08h30m–08h55m; S2: 13 March 2015 08h30m–09h00m; S3: 17 March 2013 20h50m–21h20m; and S4: 17 March 2015 16h30m–17h00m. The data sets as maps with evenly grid in ASCII files are available on request from the authors. If our data is to be used in publications or research papers, then please provide a link to this article.
